# Musculocartilaginous structure of the external ear in dromedary camels with special reference to auricular nerve blocks

**DOI:** 10.3389/fvets.2025.1689511

**Published:** 2025-10-31

**Authors:** Gamal Mounir Allouch, Fahad Abdullah Alshanbari, Madeh Sadan

**Affiliations:** ^1^Department of Medical Biosciences, College of Veterinary Medicine, Qassim University, Buraydah, Saudi Arabia; ^2^Department of Clinical Sciences, College of Veterinary Medicine, Qassim University, Buraidah, Saudi Arabia

**Keywords:** animals, anatomy, camels, ears, diagnostic imaging, nerve block

## Abstract

**Aim:**

Understanding the external ear’s anatomy and the ear nerve blocks (particularly in the external acoustic meatus) is crucial for facilitating effective ear movement and treating clinical cases requiring an ear nerve block. The purpose of this study was to describe the anatomical cartilages and muscular structures of the outer ear and to investigate the appropriate anatomical location of the major nerves supplying the ear in dromedary camels.

**Methods:**

The study was conducted on 12 adult male and female dromedary camel heads of different breeds, obtained from the Buraydah slaughterhouse. The standard dissection technique was employed by placing the samples in a 10% formalin solution before dissecting them using approved dissection tools.

**Results:**

The findings demonstrated that the auricle of camels has a particular structure. The muscles were organized into four groups: rostral, dorsal, ventral, and caudal. In addition, three cartilages were identified, along with the anatomical location of the auricular nerves. Based on anatomical and ultrasonographic landmarks, the injection sites for the internal and great auricular nerve blocks were determined at the lateral side of the base of the auricular cartilage and at the base of the pinna on its caudal side, respectively.

**Conclusion:**

The study provides detailed information about the ear pinna, cartilages, and muscles of the camel, with a particular focus on the auricular nerve block method. These findings can serve as a guide future for clinical and surgical procedures involving the camel’s ear.

## Introduction

The ear is a sensory organ with unique abilities for listening and maintaining balance (1). Together with the external auditory canal, the ear collects sound waves and transmits them to the tympanic membrane (2). The anatomical structure of the ear is divided into three parts: the outer ear, middle ear, and inner ear (3, 4). Therefore, the anatomy of the ear has been well described in many mammalian species, including the dromedary camel (5–8).

The shape of the outer ear in the majority of domestic animals is determined by the ear cartilage, which provides it with a permanent firmness (9). The pinna’s skin is thin and tightly attached to the cartilage’s periosteum (9). The auricle has many features that make it particularly easy to be evaluated by ultrasound (10). In the majority of domesticated animals, the three cartilages of the pinna are as follows: the auricular, the scutiform, and the annular cartilages (5). One exception of this is dogs, whose ear cartilages consist of the auricular and annular cartilages (11). Moreover, the pinna in canines is remarkably varied, exhibiting variations in shape and size based on the breed (12).

The mobility of the outer ear is controlled by several auricular muscles. These muscles attach at the base of the ear to the skull bones and the dorsal cervical region adjacent to the ear (3). The auricular muscles differ between and within species, and they are controlled by the intermedius facial nerve (4). The auricular muscles are divided into extrinsic and intrinsic groups. The middle ear consists of the tympanic cavity, the auditory ossicles, and the eustachian tube. The tympanic cavity is located within the petrous temporal bone (3). The auditory ossicles are attached to the wall of the tympanic cavity through several ligaments and mucosal folds. The boundary between the middle and inner ear is the oval window. The eustachian tube connects the tympanic cavity to the nasopharynx, which marks the beginning and end of the eustachian tube, respectively. The inner ear is located within the petrous temporal bone (13). The inner ear contains the membranous labyrinth, which is surrounded by the bony labyrinth. The membranous labyrinth is a network of fluid-filled membranous sacs. The fluid within these sacs is known as endolymph. It is the movement of the endolymph that stimulates the sensory cells within the membranous wall.

The objective of this study was to describe the anatomical structure of the outer ear cartilages and muscles and to locate the internal and great auricular nerve blocks to facilitate the examination of the external auditory canal in dromedary camels.

### Ethical statement

The study protocol was approved by the Laboratory Animal Control Guidelines of Qassim University, which conform to the Guide for the Care and Use of Laboratory Animals of the National Institutes of Health (NIH) in the United States (NIH publications No. 86 to 23, revised 1996).

## Materials and methods

This study used 12 healthy camel heads of different ages (1–3 years) and both sexes. The specimens were obtained from the Buraydah slaughterhouse in the Qassim region of Saudi Arabia. The samples were preserved in a 10% formalin solution for 1 week and subsequently rinsed with water. The heads were dissected to investigate the morphological and anatomical structure of the cartilages and ligaments. In addition, to study the blood vessels, red-dyed latex was injected into the common carotid artery to examine the blood supply to the auricle, and nerve staining was performed to study the nerve supply of the auricular cartilage. The injection sites for the internal and great auricular nerves were shaved and thoroughly cleaned with warm water and soap. The head was secured in a suitable position for the injection of each nerve. The injection of the nerves was performed by an experienced veterinary surgeon (MS) using a real-time scanner (Sony scan, Japan) equipped with 3.5 and 7.5 MHz sector transducers. Using ultrasound guidance, the needle was positioned at approximately a 75° angle to the skin toward the targeted nerve site, based on the anatomical landmarks of the lateral side of the base of the auricular cartilage and the base of the pinna on its caudal side, corresponding to the internal and great auricular nerves, respectively, as described by Ali et al. (14).

## Results

### The pinna (auricle)

**Figure 1:**
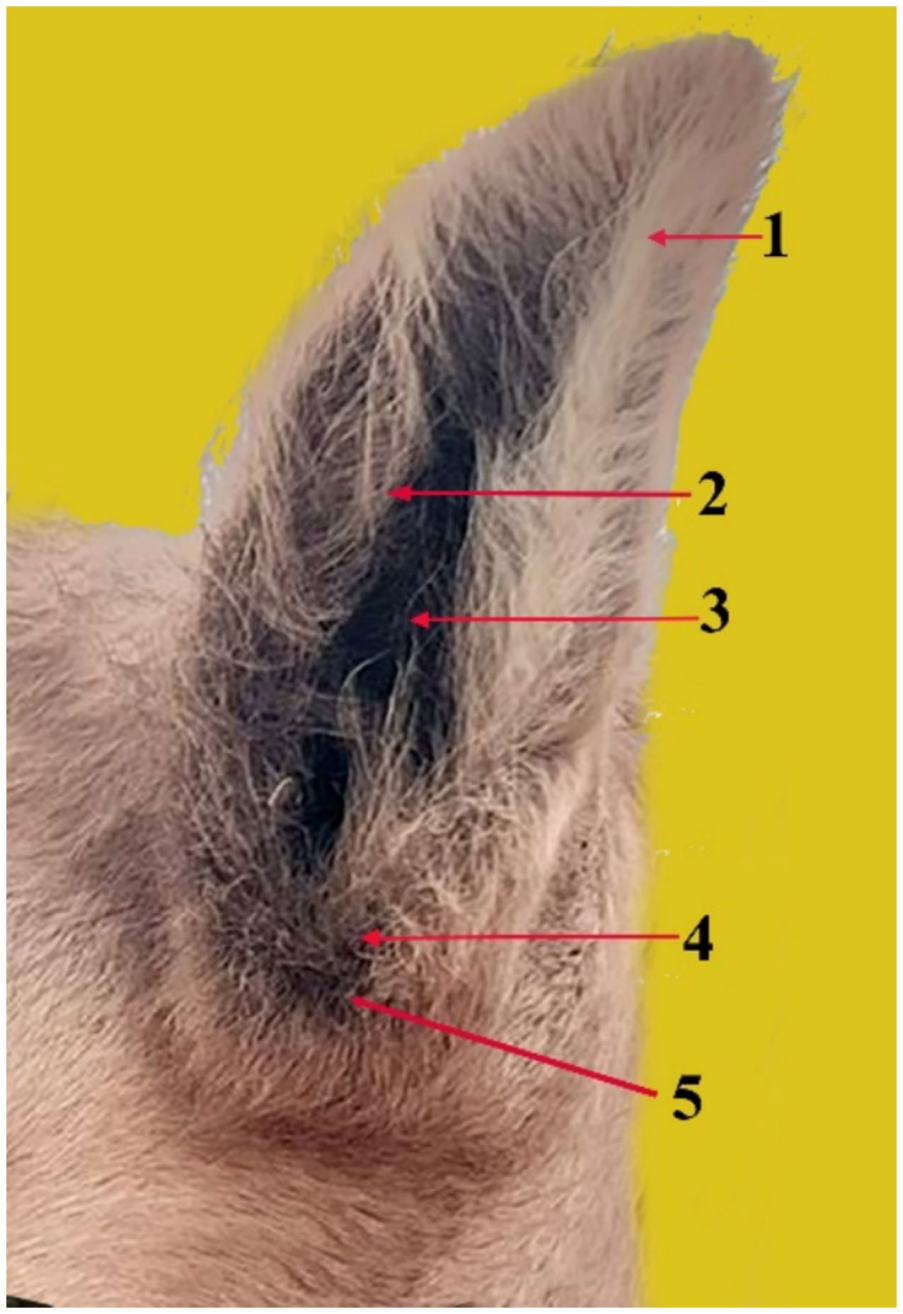
The pinna (auricle) and its components in the dromedary camel (1), showing the antihelix ridge (2), the antihelix fossa (3), the tragus (4), and the antitragus notch (5).

**Figure 2:**
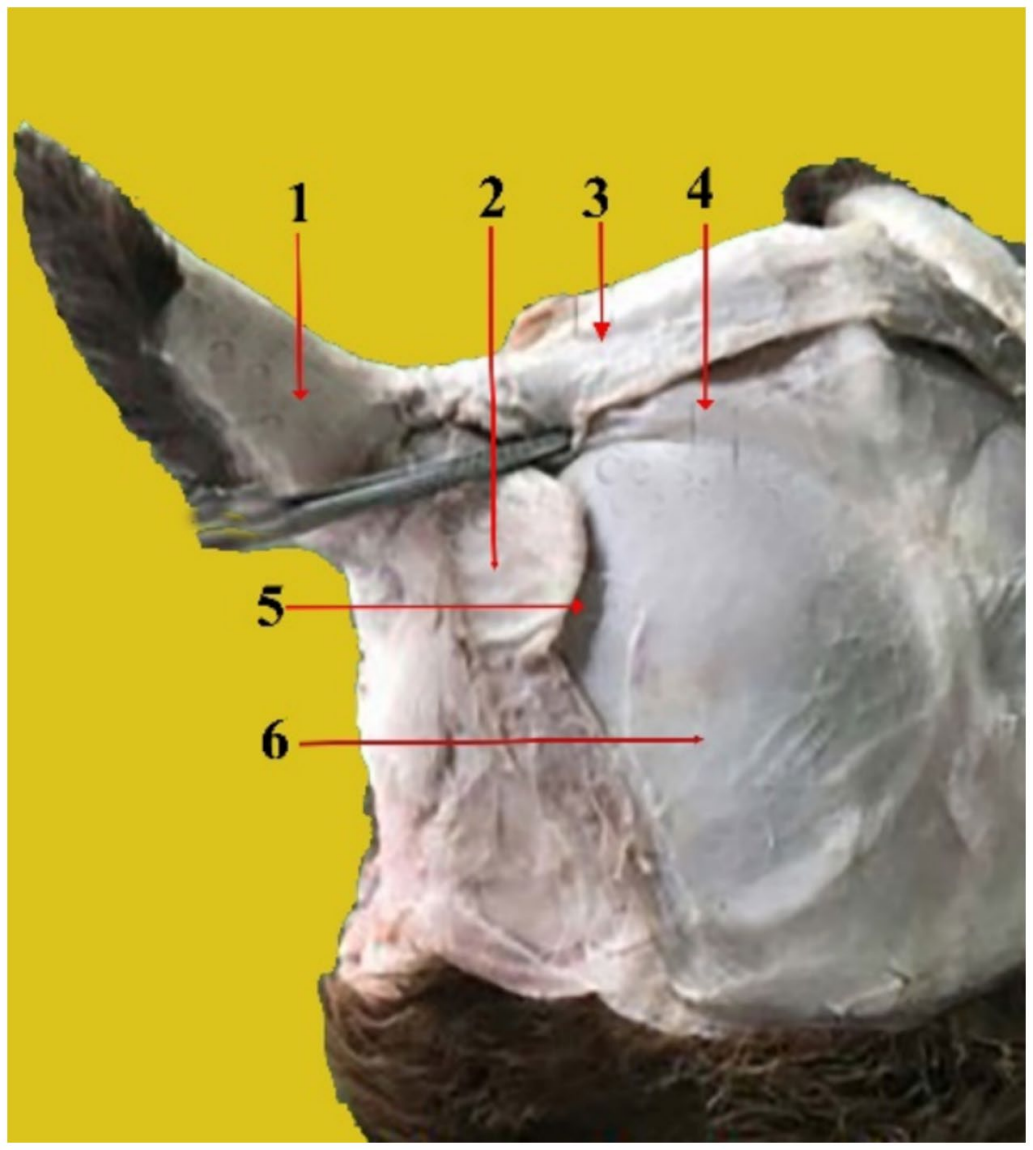
Caudal view of the dissected camel head showing the auricle cartilage (1), scutiform cartilages (2), Interscutular muscle (3), cervico-scutolar muscle (4), deep scutulo-auricular muscle (5), and temporal muscle (6).

The tip of the pinna points dorsolaterally, whereas the base of the pinna is a cartilaginous projection located on both sides of the caudal section of the skull (Figure 1). The outer ear is composed of a thin layer of flexible cartilage covered by skin with hair. The skin is delicate and has tiny hairs that attach it to the ear cartilage. It is intimately related to both the external auditory canal’s opening and the area surrounding the muscles. The auricle has lateral beveling and a funnel-like shape. The distal and apical portions are smaller than the central portion. It features two surfaces and two boundaries. The two boundaries are medial and lateral; the external margin is concave ventrally and convex dorsally, whereas the medial margin is larger than the lateral and convex. There are two surfaces: rostral and caudal. The concave, hair-covered rostral surface of the auricle pinna features uncomplicated and noticeable long grooves known as antihelix grooves. The pinna’s caudal surface is convex, smooth, and hairless, as is the antihelix ridge (Figure 1, 2). The grooves approach the ear opening and form the pinna. Therefore, the concha is formed by the antihelix curving around a large and deep hollow. The tragus, a little round lump that covers part of the ear opening and marks the external edge of the ear canal orifice, is located next to the distal section of the auricle (Figure 1). The antitragus notch is located ventral to the auricle, near the entrance of the ear opening (Figure 1, 5).

### Anatomy of the outer ear cartilages

The cartilages of the outer ear consist of the auricular cartilage, scutiform cartilage, and annular cartilage. The auricular cartilage (Figure 2) is the external part of the auricle. It gives the ear a funnel-like shape. It consists of a single piece that supports the outer ear. It is joined to the skull by the cricoid cartilage.

The scutiform cartilage (Figure 2, 2) is a disc-shaped structure that connects to the pinna and the temporal muscles on the skull in the temporal region. It lies cranial to the ear cartilage and has two surfaces: superficial and deep. It also has two borders (cranial and caudal). The superficial facet is slightly convex. The deep facet is concave. On the other hand, the caudal margin is robust and broad, whereas the cranial margin is narrow and rounded.

**Figure 3:**
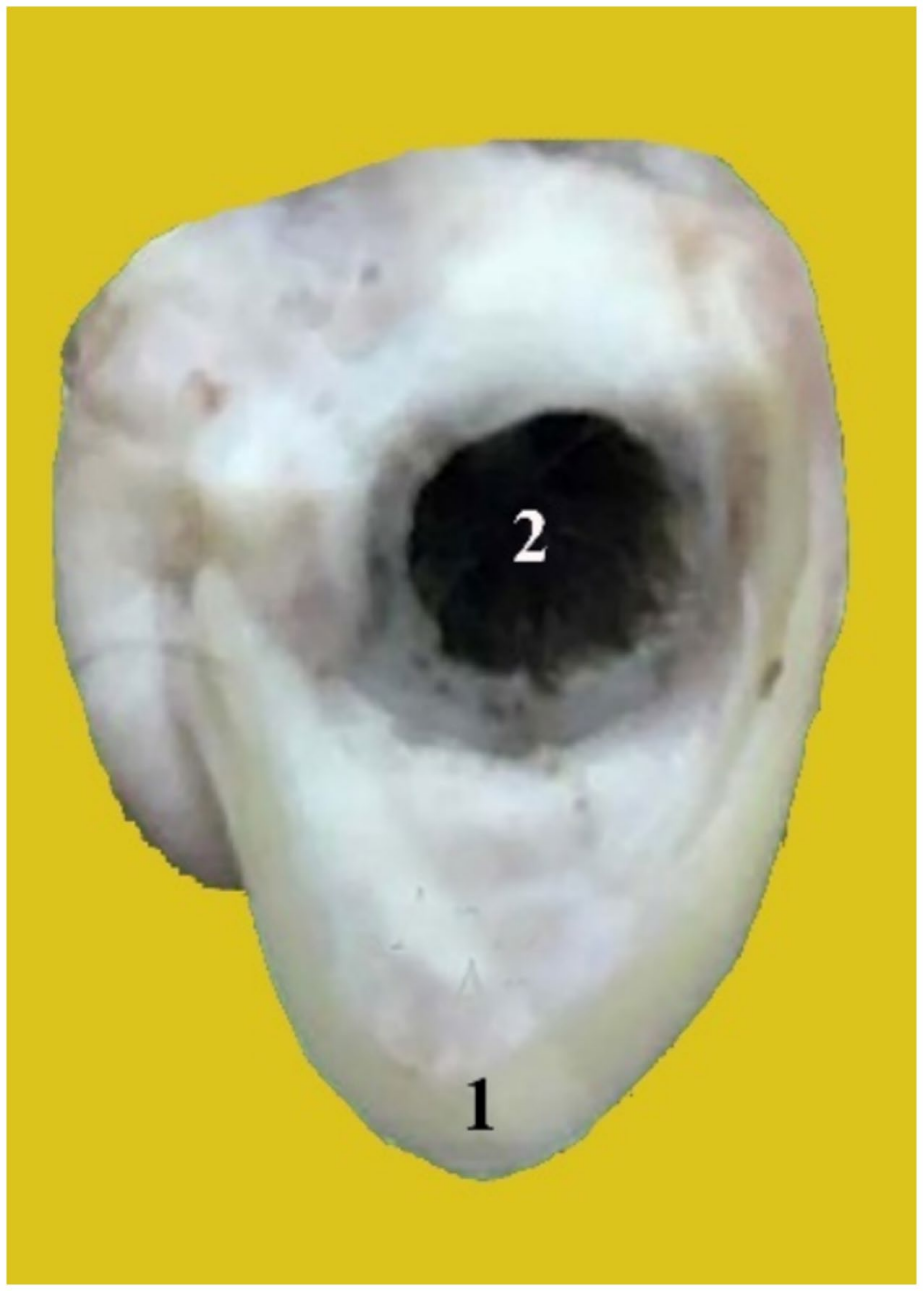
A photograph shows the annular cartilage (1) and the external acoustic meatus (2).

The annular cartilage (Figure 3, 1) is situated near the base of the ear’s cartilage. It is shaped like a ring and is rounded. It is folded into a small tube with a diameter of approximately 1 cm and connected medially by elastic tissue. There are two crura in it: the left and right crus. It provides a cartilaginous section that forms the opening of the external auditory canal and contacts the distal end of the ear cartilage.

### Anatomy of the outer ear muscles

**Figure 4:**
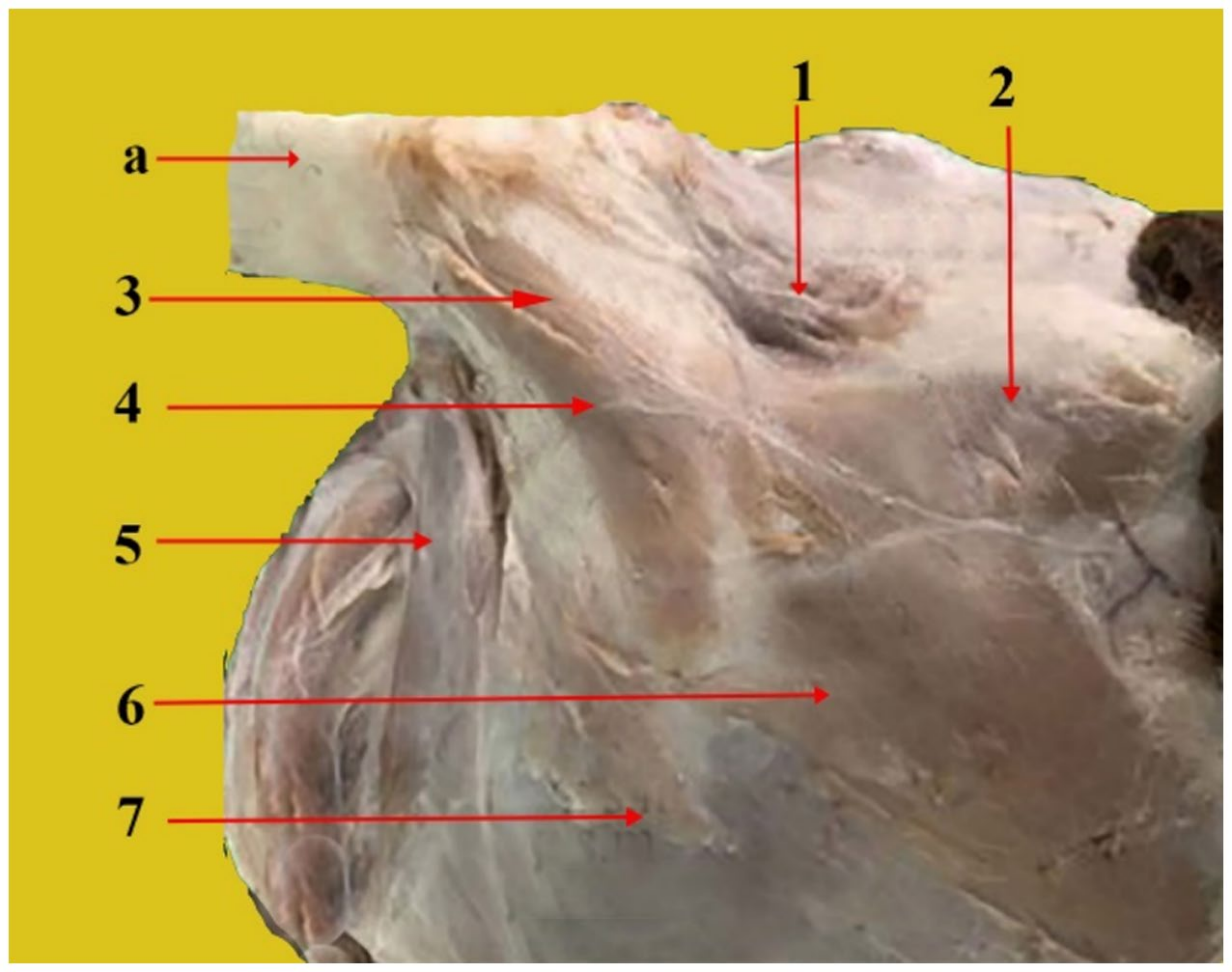
Rostral, dorsal, and cranial views of a dissected dromedary camel head showing the different muscles that are attached to the auricular cartilage. From the top to the bottom of the picture: Auricle cartilage (a), superficial scutulo-auricular muscle (1), fronto-scutular muscle (2), middle scuto-auricular muscle (3), dorsal scuto-auricular muscle (4), parotidoauricular muscle (5), zygomatico-auricular muscle (6), and zygomatico-scutular muscle (7).

**Figure 5:**
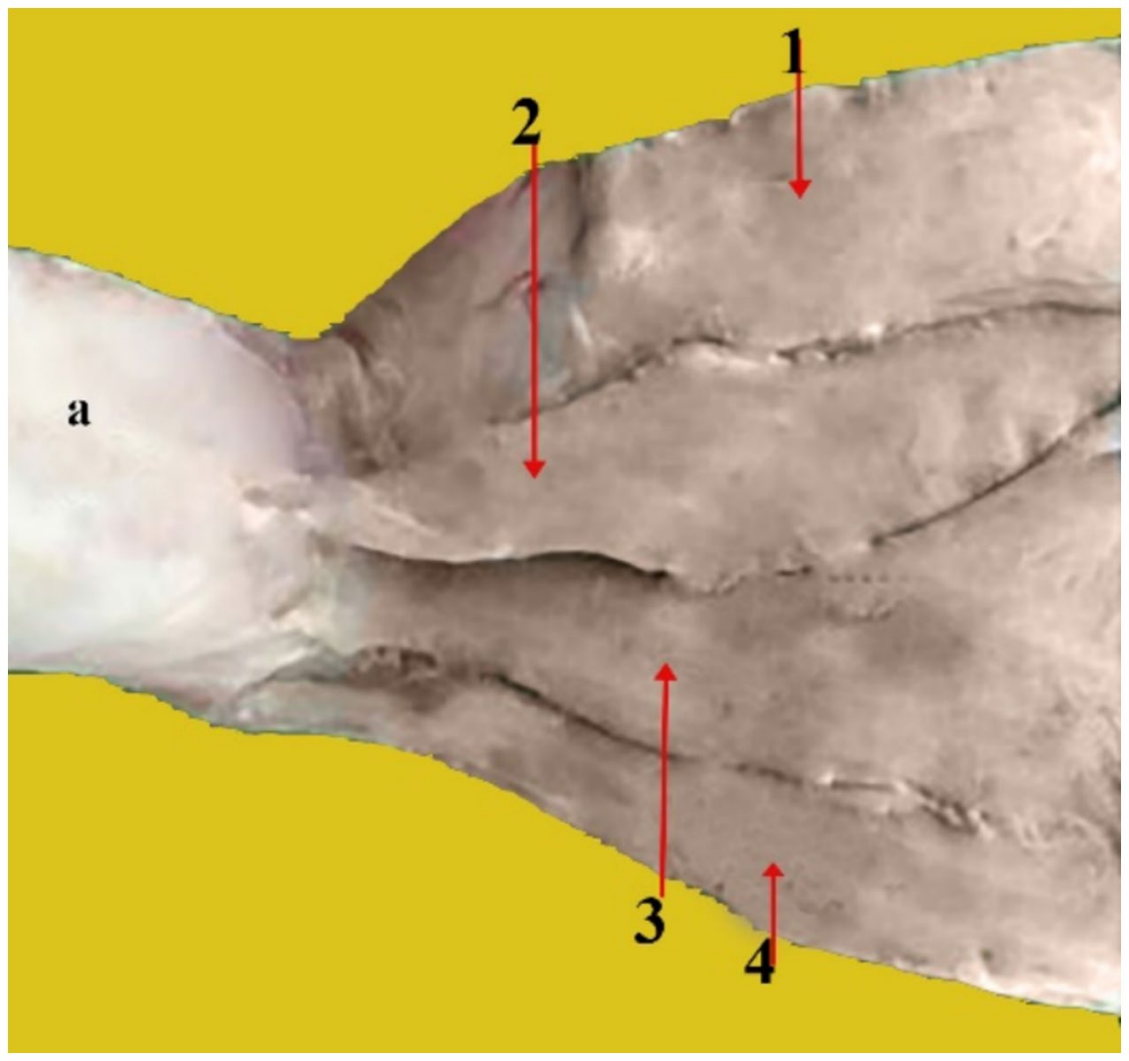
Caudal view of a dissected camel head showing the caudal auricular muscles: Auricular cartilage (a), Interscutular muscle (1), superficial cervico-auricular muscle (2), middle cervico-auricular muscle (3), and deep cervico-auricular muscle (4).

**Figure 6:**
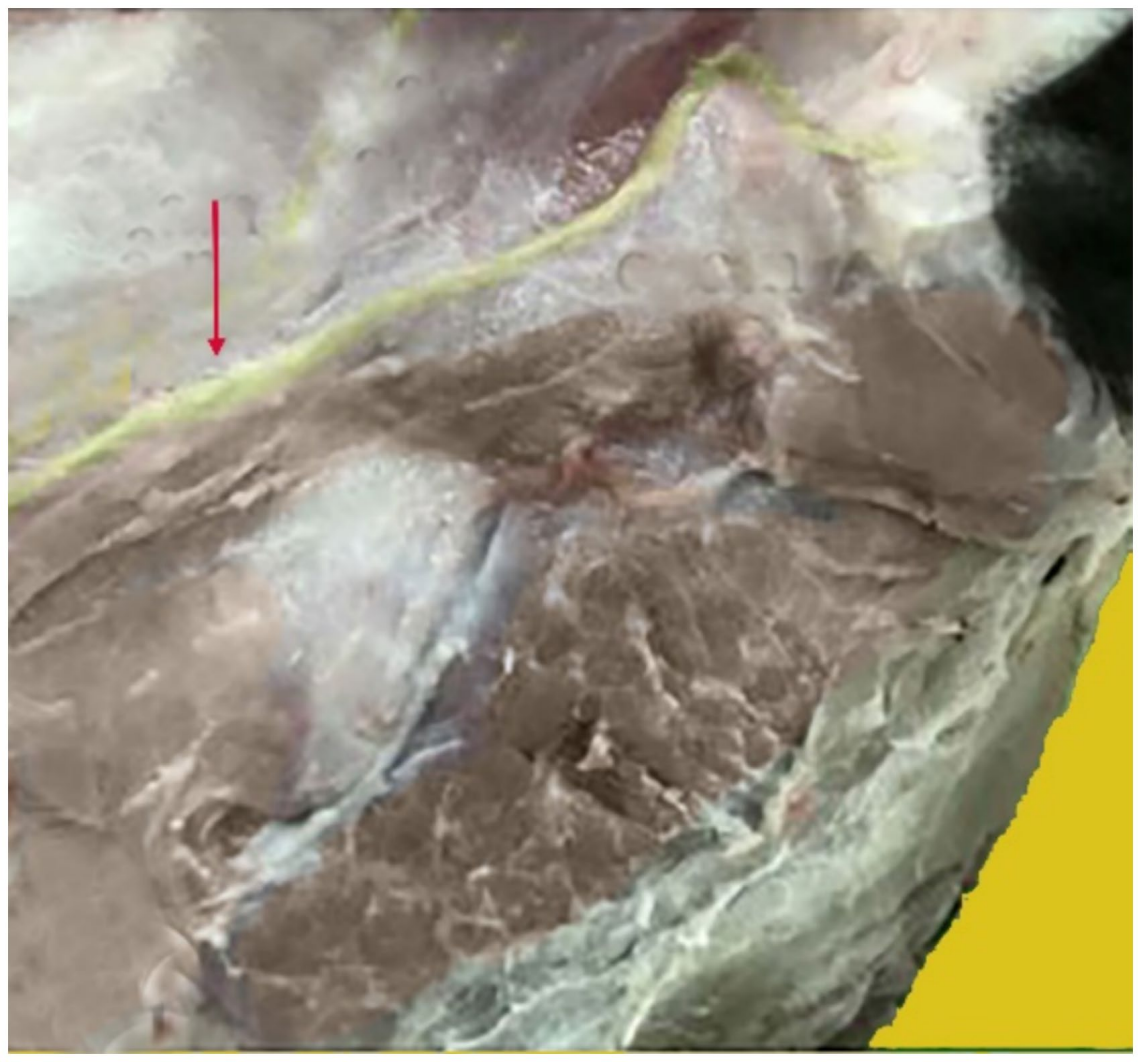
Anatomical position of the greater auricular nerve. The nerve shown in yellow, indicated by the red arrow, supplies the camel auricle.

**Figure 7:**
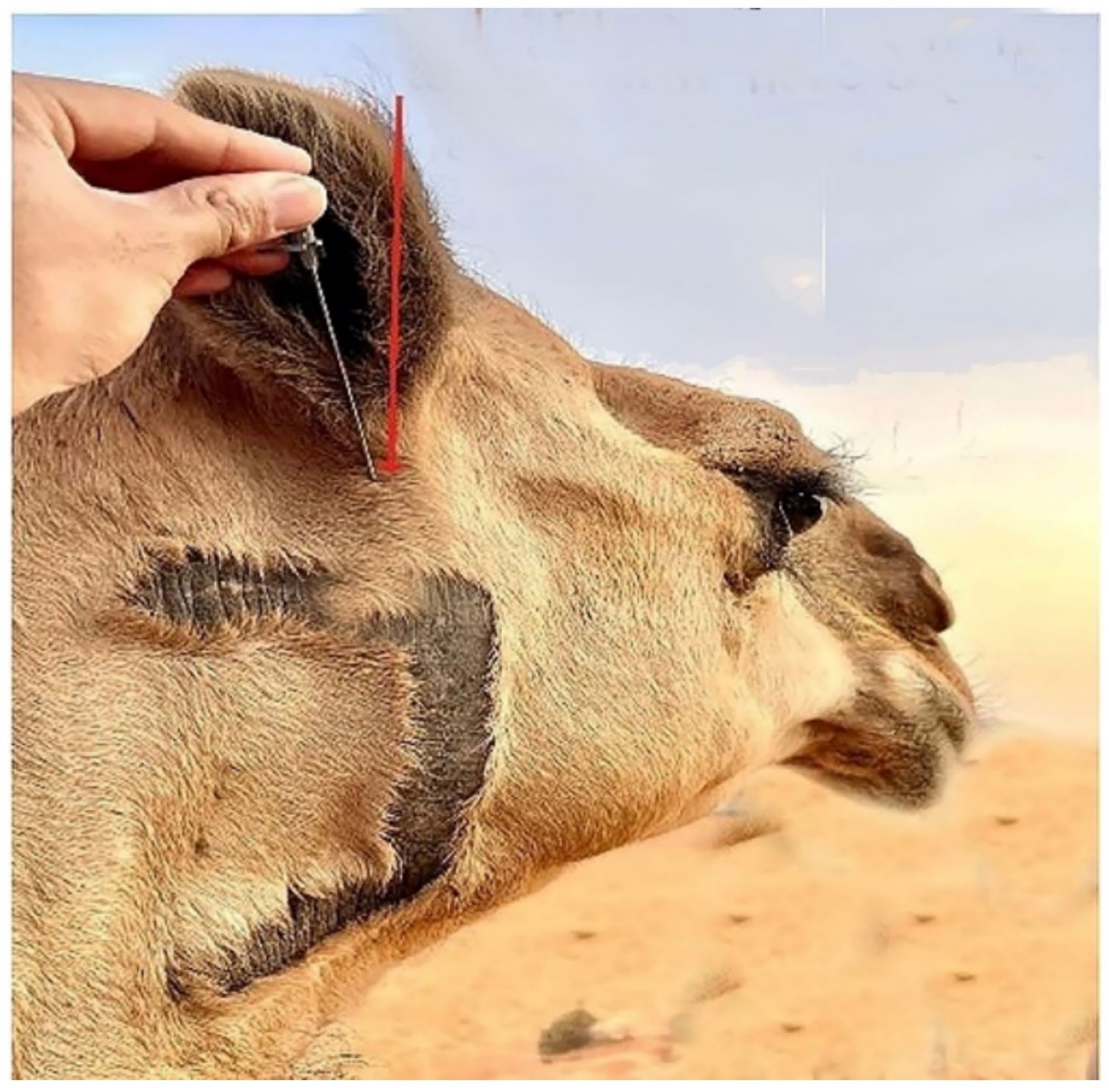
An image displaying the location site of the internal auricular nerve block in the dromedary camel (Red arrow).

We classified the outer ear muscles based on their location into four muscle groups:

1. Rostral auricular muscles

- Superficial scutulo-auricular muscle (Figure 4, 1): It has a nearly circular shape. The dorsal side of the scutiform cartilage is its origin, whereas the insertion is in the distal part of the auricular cartilage. The interscutular muscle covers a portion of it. It extends from the caudal marginal cartilage to the cranial edge of the auricle.
- Deep scutulo-auricular muscle (Figure 2, 5): Its form is comparable to that of the preceding muscle. It is well developed and has a circular form. It originates from the scutiform cartilage’s deep surface. It is located on the scutiform cartilage’s deep aspect. Its fibers extend to the caudal portion of the ear cartilage and run caudally.
- Frontoscutular muscle (Figure 4, 2): It is shaped like a trapezoid, and its smaller base is positioned rostrally. It originates from the temporal line and the frontal bone’s zygomatic process. It inserts into the rostral edge of the scutiform cartilage and is divided into frontal and temporal sections.
- Zygomatico-auricular muscle (Figure 4, 6): It is shaped like a rectangle. It originates at the rostrolateral boundary of the scutiform cartilage and extends directly from the arch of the zygomatic bone. It inserts on the lateral side of the auricular cartilage, extends along the zygomatic bone, and is partially fused ventrally with the superficial scutulo-auricular muscle. It can extend to the auricular cartilage.
- Zygomatico-scutular muscle (Figure 4, 7): It is situated ventrally beneath the zygomatic auricular muscle. It is shaped like a triangle. It emerges from the frontal bone’s base of the zygomatic process and attaches itself to the scutiform cartilage’s rostral border.

2. Dorsal auricular muscles:

- Interscutular muscle (Figures 2, 3 and 5, 1): It originates from the parietal bone. It is a long and thick muscle. It connects the left and right scutiform cartilages, which extend from the auricular cartilage to the temporal line and the external sagittal crest of the parietal bone.
- Cervico-scutolar muscle (Figure 2, 4): It has a triangular, arched shape. It originates from the sagittal crest at the base of the occipital bone.
- Dorsal scuto-auricular muscle (Figure 4, 4): It originates from the scutiform cartilage, and the insertion is on the rostrolateral part of the ear auricle.
- Middle scuto-auricular muscle (Figure 4, 3): It starts from the scutiform cartilage to reach the center of the base of the pinna. The scutiform and auricular cartilages are connected by these muscles.

3. Ventral auricular muscle:

The ventral auricular muscle has only one muscle, the parotidoauricular muscle (Figure 4, 5). It is a superficial muscle. It is a thin and slender muscle that resembles a ribbon. It covers the parotid gland’s lateral side. It starts on the ventral side of the parotid fascia, extends ventrally and then joins the ventrolateral portion of the auricular cartilage.

4. Caudal auricular muscles: These muscles are distinct and highly developed. The scutulum is pulled caudally.

- Superficial cervico-auricular muscle (Figure 5, 2): It is rectangular in shape and thin. It is located superficial to the other outer auricular muscles. It starts from the fascia lamina of the atlas near the vertical area of the occipital ligament and extends from the dorsal region of the neck almost to the pinna, the base of the auricular cartilage’s caudal surface where it is inserted.
- Middle cervico-auricular muscle (Figure 5, 3): It has a distinct muscular belly and resembles a spindle. At its origin, it fuses with the deep cervico-auricular muscle. It is located in the space between the deep and superficial cervico-auricular muscles. It begins from the Atlantal fascia near the ventral surface of the superficial cervico-auricular muscle and the lamina portion of the nuchal ligament, and it inserts at the base of the caudal surface of the auricular cartilage beneath the superficial cervico-auricular muscle.
- Deep cervico-auricular muscle (Figure 5, 4): It is located below the middle cervico-auricular muscle. It starts from the Atlanta fascia near the middle cervical muscle’s vertical portion of the nuchal ligament. Its insertion is on the posterior aspect of the auricular cartilage, beneath the middle cervico-auricular muscle.

The names, origins, and insertions of these muscles are summarized in Table 1.

**Table 1:** Outer ear muscles and their origins and insertions.

Muscle name	Origin	Insertion
Superficial scutulo-auricular muscle	The scutiform cartilage	The distal part of the auricular cartilage
Deep scutulo-auricular muscle	Scutiform cartilage’s deep surface	Caudal portion of the ear cartilage, extending caudally
Fronto-scutular muscle	The temporal line and the frontal bone’s zygomatic process	The rostral edge of the scutiform cartilage
Zygomatico-auricular muscle	The rostrolateral boundary of the scutiform cartilage	The lateral side of the auricular cartilage
Zygomatico-scutular muscle	The zygomatic process	The scutiform cartilage’s rostral border
Interscutular muscle	The parietal bone	The auricular cartilage
Cervico-scutolar muscle	The sagittal crest	The auricular cartilage
Dorsal scuto-auricular muscle	The scutiform cartilage	The rostrolateral portion of the ear auricle
Middle scuto-auricular muscle	The scutiform cartilage	The center of the base of the pinna
Parotidoauricular muscle	The ventral side of the parotid fascia	The ventrolateral portion of the auricular cartilage
Superficial cervico-auricular muscle	The fascia lamina of the atlas near the vertical area of the occipital ligament	The base of the auricular cartilage’s caudal surface
Middle cervico-auricular muscle	Atlantal fascia	The base of the caudal surface of the auricular cartilage
Deep cervico-auricular muscle	Atlanta fascia	The posterior aspect of the auricular cartilage

### Internal and great auricular nerve blocks

The external auditory canal and the inside surface of the ear are innervated by the internal auricular nerve, a branch of the cranial facial nerve. It enters the auricle at its deepest point, running beneath the parotid gland. On the other hand, the great auricular nerve is a branch of the cervical nerve that extends subcutaneously from the second cervical vertebra to the caudal side of the base of the ear (Figure 6).

**Figure 8:**
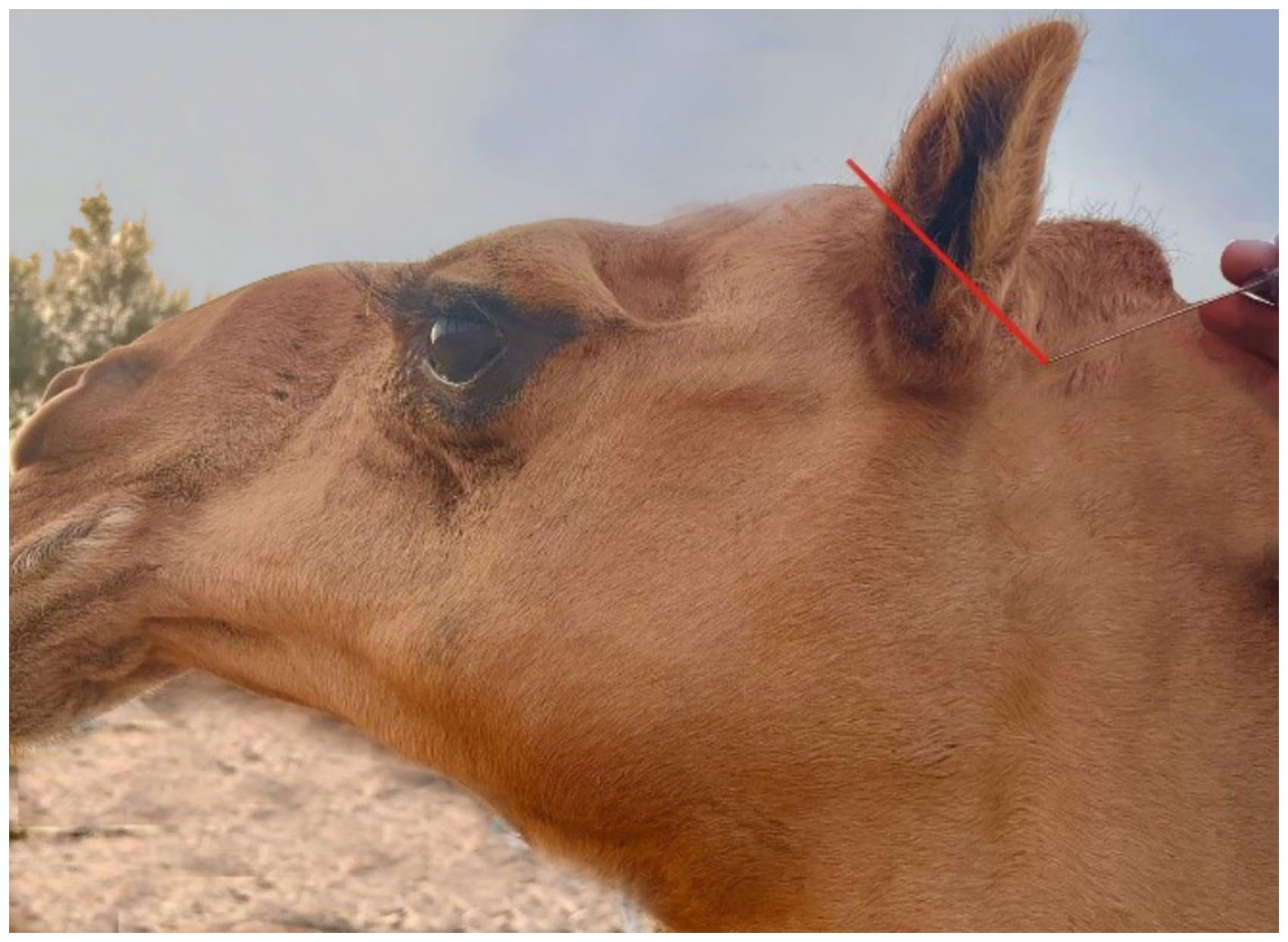
A picture showing the site of the great auricular nerve block in the dromedary camel (Red arrow).

The lateral side of the base of the auricular cartilage is palpable in response to the blockage of the internal auricular nerve (Figure 7, Red arrow). There should be a visible, tiny incision. The internal auricular nerve passes through this aperture to reach the auricular cartilage. The great auricular nerve block is located at the base of the pinna on its caudal side (Figure 8, Red arrow). When palpated, the great auricular nerve can be felt sliding beneath the fingers as it runs subcutaneously in a rostrocaudal direction.

**Figure 9:**
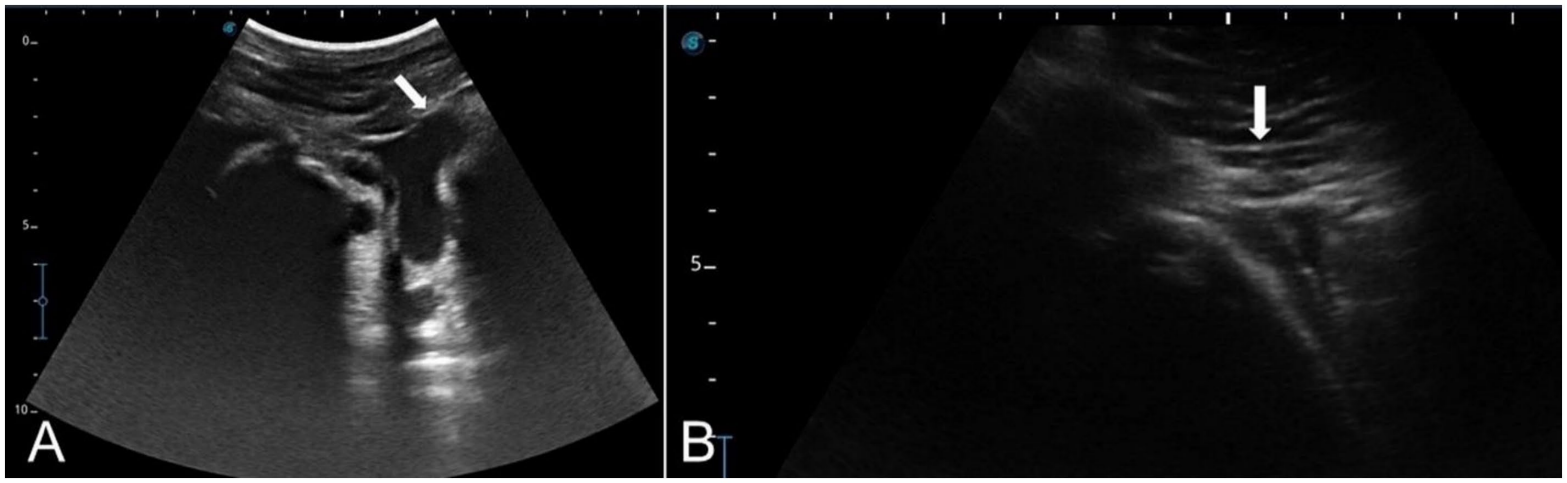
Linear ultrasound images showing the auricular nerve at the distal third of a live camel’s ear. **(A)** Great auricular nerve. **(B)** Internal auricular nerve.

Each nerve of the dromedary camel’s ear could be precisely discriminated using anatomical and ultrasonographic-guided techniques. The anatomical landmarks determining the point of needle insertion for the internal and great auricular nerves were successfully identified and confirmed by ultrasonography (Figures 9A,B). Based on the anatomical and ultrasonographic landmarks, the site of injection for the internal and great auricular nerves was approached at the lateral side of the base of the auricular cartilage and the base of the pinna on its caudal side, respectively.

### Blood supply to the auricular cartilages

**Figure 10:**
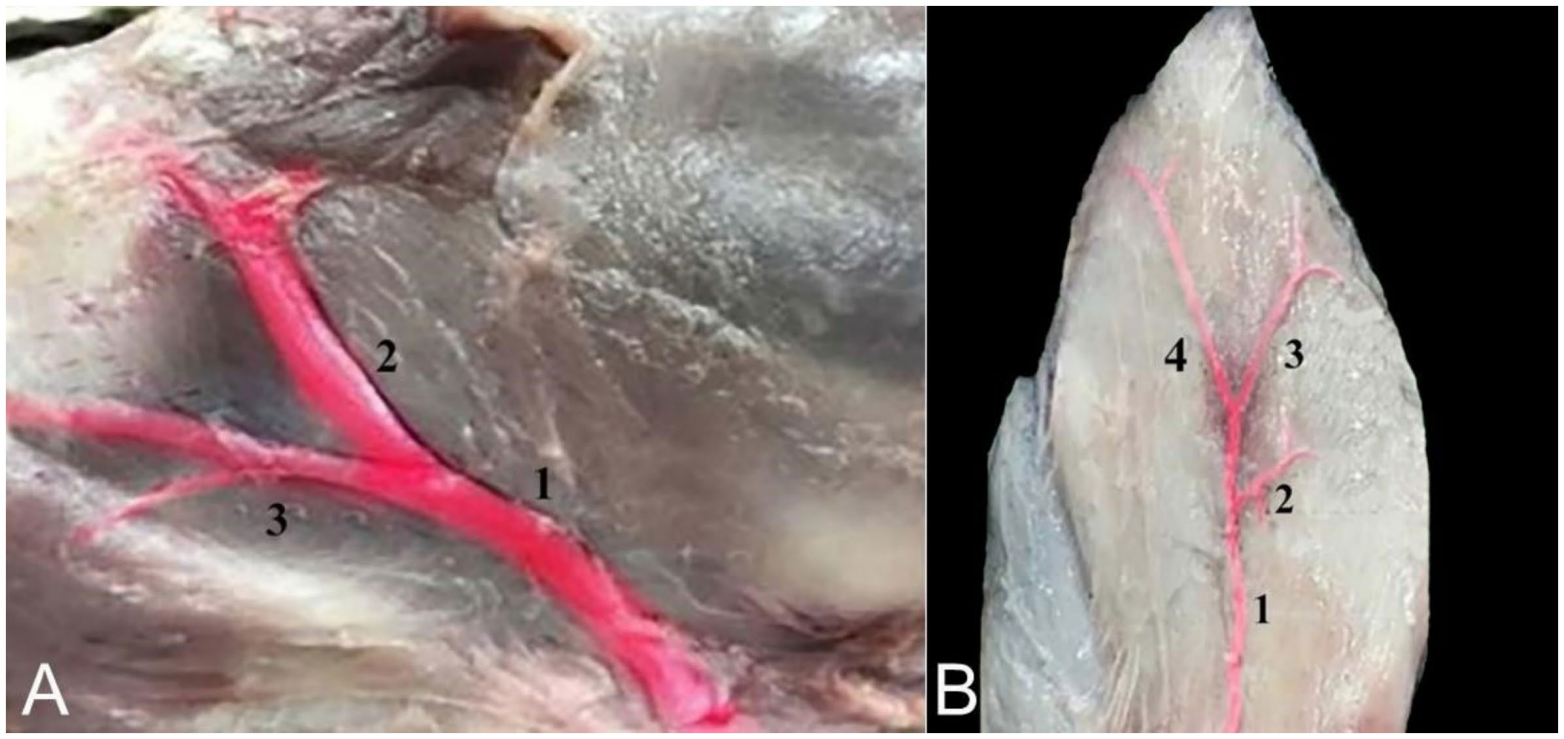
**(A)** An image showing the blood supply of the camel auricle: Superficial temporal artery (1), rostral auricular artery (2), and maxillary artery (3). **(B)** An image showing the blood supply to the cartilages of the camel ear: Caudal auricular artery (1), medial auricular branch (2), intermediate auricular branch (3), and lateral auricular branch (4).

The auricular cartilages receive their blood supply from two arteries: The caudal auricular artery, which arises directly from the external carotid artery, and the rostral auricular artery, branching from the superficial temporal artery, which itself branches from the external carotid artery. The caudal auricular artery divides into three branches on the convex side of the auricle—the medial, intermediate, and lateral auricular branches—to supply blood to the caudal side of the auricle. The venous drainage of the ear is facilitated by the caudal auricular vein, which originates from the external carotid vein. It removes blood from the lateral, intermediate, and medial auricular veins (Figure 10).

## Discussion

The outer ear pinna (auricle), cartilages, muscles, and nerve blocks were investigated in this study. The outer ear consists of the pinna (auricle), scutiform, and annular cartilages. The muscles were divided into four groups based on their locations: rostral, dorsal, ventral, and caudal muscles. In addition, the internal and great nerve blocks were identified.

The outer ear anatomy of the dromedary camel is comparable to that of other domesticated animals. The pinna (auricle) (Figure 1) has a funnel-like shape and includes antihelix ridges, which are simple, noticeable longitudinal ridges (Figure 1, 2), and antihelix fosses, which are depressions (Figure 1, 3). Similar results were observed in cattle, buffalo, deer, and sheep, where the pinna cavity is encircled by a tube made of the basal portion of the auricle cartilage (15, 16).

The distal part of the pinna forms the tragus. The tragus is formed by the proximal portion of the tragus margin rolling caudolaterally (Figure 1, 4). This result is in line with what has been described in other domestic animals (15, 17, 18). On the other hand, the comparable region of the antitragus border forms a well-developed cartilaginous plate (Figure 1, 5). Similar findings have been reported in sheep, dogs, and rabbits (19–21).

We showed that the external ear of the dromedary camel consists of three cartilages: Auricular (Figures 2, 1, 4, a, 5, a), scutiform (Figure 2, 2), and annular cartilages (Figure 3, 1). Our results are in agreement with findings reported in domestic animals, such as buffaloes and cattle (15, 17, 18). In addition, our results differ from those reported in dogs, which have only two cartilages—the auricular cartilage and the annular cartilage (11). The auricular cartilage is flattened and consists of elastic cartilage, which contributes to the shape of the outer ear in the dromedary camel (Figures 2, 1, 4, a, 5, a). These results are consistent with findings reported in other domesticated animals (17, 18). However, the auricular cartilage in dogs varies in shape, size, and morphology based on breeds (11).

The scutiform (Figure 2, 2) and annular cartilages (Figure 3, 1) provide support for the pinna. Both are made up of disc-shaped lamellae. In the dromedary camel, the scutiform cartilage has a diamond-shaped plate (Figure 2, 2). However, it has a triangular plate in cattle and buffalo (22, 23). The annular cartilage is a quadrangular plate shaped into an approximately three-quarter ring, wrapped in elastic tissue to form a small tube. It resembles a circular ring that is joined medially by elastic fibers (Figure 3, 1). Our results are in agreement with findings reported about the annular cartilage in other domesticated animals (15–18).

The external auditory meatus is oval, large, curved, and directed ventrally before angling medio-rostrally (Figure 3, 2). It consists of two sections, the cartilage and the osseous. In the dromedary camel, this structure is relatively long, similar to findings reported in Bactrian camels (24). The external auditory meatus extends from the outer ear to the tympanic membrane (eardrum membrane). The outer part of the ear canal is the annular cartilage, whereas the inner section of the canal is the internal acoustic meatus, which passes through the temporal bone (13, 25). The anatomical significance of the external acoustic meatus lies in its role in facilitating easy examination of the tympanic cavity (26, 27). The width and length of the external acoustic canal provide an explanation for why infections, eardrum lesions, and epidemics are prevented from spreading quickly.

The movement of the ear is caused by several ear muscles. These muscles are divided into four muscle groups: rostral (five muscles), dorsal (four muscles), ventral (one muscle), and caudal (three muscles) (Table 1). These muscles are identical to those found in domestic animals in their origin, position, orientation, and insertion (3, 5). While the anatomy of these muscles has been well described in other mammalian species, this is the first detailed report on the dromedary camel’s outer ear muscles. The auricular muscles are widely believed to have minimal functional value (28). However, these ear muscles play a crucial role, as they allow the animal to move its ears toward surrounding sounds (13, 29).

The nerves in a camel’s ear are very important because they provide both motor and sensory functions to the skin, muscles, and cartilage of the ear. However, there are no detailed descriptions regarding the nerve block with anatomical landmarks of the camel ear in the existing literature. Our findings revealed that the camel’s ear receives its nerve supply through two nerves: the internal auricular nerve and great auricular nerve. These findings are consistent with those reported by Ali et al. (14). All injection requirements were highly accurate when compared to blind techniques, particularly when applying the ultrasound-guided injection technique to the output of nerve blocks.

Needle insertion using the blind technique relies on palpation of surface anatomical landmarks. This results in a limited ability to locate the anatomical site properly for needle insertion and can lead to incorrect positioning of the needle and insufficient nerve block. Moreover, the US injection technique is a unique imaging method that is simple to perform, cost-effective under working conditions, and carries little risk. US-guided techniques can be easily integrated into a diagnostic or therapeutic procedure, especially for animals with ear injuries. Moreover, our findings are in line with those of Alloush et al. (30).

As otitis occurs in the external ear of the dromedary camel (31, 32), anesthetizing the internal and great auricular nerves can help veterinarians perform ear examinations and surgeries on the ear and its surrounding area. Desensitization of the auricle for short procedures can be achieved simply and effectively by blocking the internal and great auricular nerves, which has been successfully achieved in horses and dogs (33–35).

## Conclusion

This study investigated the anatomical structure of the outer ear in the dromedary camel. The outer ear consists of the pinna and its visual structures and the three cartilages of the outer ear. Additionally, the anatomy and locations of the associated muscles and the positions of the internal and great auricular nerve blocks were examined. The overall outcome of this study provides knowledge about the anatomy of the outer ear and supports veterinarians in performing local anesthesia on the dromedary camel’s ear.

## Data Availability

The original contributions presented in the study are included in the article/supplementary material, further inquiries can be directed to the corresponding author.
